# Towards a "Biography" of Population Health Data about Substance Use Disorders: Qualitative Approaches to Communicating Context

**DOI:** 10.23889/ijpds.v9i5.2592

**Published:** 2024-09-18

**Authors:** JJ Morgan, SS Nolan, JJ Shoveller, KK McGrail

**Affiliations:** 1 University of British Columbia; 2 BC Centre on Substance Use; 3 Dalhousie University; 4 Health Data Research Network Canada

## Background

Understanding contexts of data production is necessary for using data accurately and ethically. “Biographies of data"" have developed as a complement to existing metadata as a way to critically examine and communicate social structures and power dynamics encoded in data. This project is focused on developing a “biography” of diagnostic codes, used to identify substance use disorders (SUD) in population health datasets. People with SUD regularly experience stigma and exclusion, particularly from healthcare settings.

## Methods

Qualitative semi-structured interviews were conducted in 2024 with ""producers"" and “managers” of administrative data, including physicians and health information professionals, in British Columbia, Canada. The interviews explored processes involved with coding and billing, and the meaning of diagnostic codes from the perspective of those involved. This includes considerations of the population, social, and geographic factors, and implications of coding as inputs to decision-making. Reflexive thematic analysis was used to identify broad themes and report findings.

## Results

Although data collection and analysis is ongoing, emerging findings have revealed structural, institutional, and social-level processes that influence coding decisions, as well as potential implications on research, policy making, and knowledge production about SUD.

## Conclusions

Addressing the complexities of SUD and the overdose crisis will require a variety of data sources and approaches, and linked population health and administrative data will continue to be one important resource. Through better understanding the contexts and processes that inform the production and management of SUD-related population data, we will shed light on the ethical and social implications of their use.

